# A cross-sectional assessment of PRRSV nucleic acid detection by RT-qPCR in serum, ear-vein blood swabs, nasal swabs, and oral swabs from weaning-age pigs under field conditions

**DOI:** 10.3389/fvets.2023.1200376

**Published:** 2023-08-10

**Authors:** Onyekachukwu H. Osemeke, Guilherme A. Cezar, Rodrigo C. Paiva, Daniel C. A. Moraes, Isadora F. Machado, Edison S. Magalhaes, Ana Paula S. Poeta Silva, Mafalda Mil-Homens, Li Peng, Swaminathan Jayaraman, Giovani Trevisan, Gustavo S. Silva, Phillip C. Gauger, Daniel C. L. Linhares

**Affiliations:** ^1^Fieldepi, Iowa State University College of Veterinary Medicine, Ames, IA, United States; ^2^Veterinary Diagnostic and Production Animal Medicine Department of the College of Veterinary Medicine, Iowa State University, Ames, IA, United States

**Keywords:** PRRSV, surveillance, swine, RT-qPCR, swab, pool, weaning, serum

## Abstract

**Introduction:**

The porcine reproductive and respiratory syndrome virus (PRRSV) continues to challenge swine production in the US and most parts of the world. Effective PRRSV surveillance in swine herds can be challenging, especially because the virus can persist and sustain a very low prevalence. Although weaning-age pigs are a strategic subpopulation in the surveillance of PRRSV in breeding herds, very few sample types have been validated and characterized for surveillance of this subpopulation. The objectives of this study, therefore, were to compare PRRSV RNA detection rates in serum, oral swabs (OS), nasal swabs (NS), ear-vein blood swabs (ES), and family oral fluids (FOF) obtained from weaning-age pigs and to assess the effect of litter-level pooling on the reverse transcription-quantitative polymerase chain reaction (RT-qPCR) detection of PRRSV RNA.

**Methods:**

Three eligible PRRSV-positive herds in the Midwestern USA were selected for this study. 666 pigs across 55 litters were sampled for serum, NS, ES, OS, and FOF. RT-qPCR tests were done on these samples individually and on the litter-level pools of the swabs. Litter-level pools of each swab sample type were made by combining equal volumes of each swab taken from the pigs within a litter.

**Results:**

Ninety-six piglets distributed across 22 litters were positive by PRRSV RT-qPCR on serum, 80 piglets distributed across 15 litters were positive on ES, 80 piglets distributed across 17 litters were positive on OS, and 72 piglets distributed across 14 litters were positive on NS. Cohen's kappa analyses showed near-perfect agreement between all paired ES, OS, NS, and serum comparisons (). The serum RT-qPCR cycle threshold values (Ct) strongly predicted PRRSV detection in swab samples. There was a ≥ 95% probability of PRRSV detection in ES-, OS-, and NS pools when the proportion of positive swab samples was ≥ 23%, ≥ 27%, and ≥ 26%, respectively.

**Discussion:**

ES, NS, and OS can be used as surveillance samples for detecting PRRSV RNA by RT-qPCR in weaning-age pigs. The minimum number of piglets to be sampled by serum, ES, OS, and NS to be 95% confident of detecting ≥ 1 infected piglet when PRRSV prevalence is ≥ 10% is 30, 36, 36, and 40, respectively.

## 1. Introduction

Despite advances in the knowledge of porcine reproductive and respiratory syndrome virus (PRRSV) ecology, laboratory investigation techniques, prevention, control, and elimination strategies, the virus continues to pillage the global swine industry, causing wanton productivity losses (1–4). Active PRRSV surveillance, which is a crucial component of PRRSV control and elimination programs (5, 6), has gained increased adoption and participation in the United States, as evidenced by annual increases in sample submissions to major veterinary diagnostic laboratories in the United States for PRRSV investigation (7, 8).

Owing to the ecology of PRRSV (9, 10), frequent sampling of a representative proportion of a herd is needed to provide a reliable picture reliable picture of viral activity in the herd. Serum sampling is a classical method for monitoring PRRSV, but it involves a higher level of skill to collect, is invasive, and is inconvenient both for the pigs and the person performing the sampling (11). In recent years, the swine industry has leaned more toward aggregate samples for PRRSV surveillance (8). This shift may be attributed to the ease of sample collection and the representation of a larger proportion of the herd with these samples. However, individual pig samples are still very useful in PRRSV surveillance, such as in estimating PRRSV prevalence (12), measuring antibody responses (13, 14), or conducting advanced molecular diagnostic tests. Swabs are an alternative to serum for individual pig sampling and have proven to be effective in investigating PRRSV RNA through RT-qPCR; for example, ear-vein blood swabs and their pools are practical alternatives to serum for conducting PRRSV surveillance in boar studs (15). There have also been reports of the use of ear-vein blood swabs from weaning-age pigs for PRRSV surveillance in outdoor swine herds in the UK (16). A few earlier studies have revealed that PRRV RNA could be detected earlier (17–20) and more frequently (21) in nasal swabs (NS) than in serum samples. The use of oral swabs (OS) in PRRSV surveillance has also been similarly demonstrated (22, 23).

Although the collection of swabs is less invasive and more convenient to the sampler and pig than serum sampling, all swab samples do not offer equal levels of comfort to the pigs; for example, pigs are still restrained and experience discomfort when NS is collected (24, 25). OS sampling is perhaps the least invasive and most comfortable of the swab sample types, as young pigs are wont to interacting with objects using their mouths (26). As the prevalence of PRRSV in a herd decreases, the number of pigs or litters to be sampled for PRRSV monitoring/surveillance increases to maintain detection probabilities (27, 28), inadvertently elevating the costs for diagnostic tests. The dilemma of testing fewer animals or testing the required number of animals in groups (pools) is one major reason various studies have assessed the effect of pooling on the RT-qPCR detection of PRRSV RNA. The effect of pooling on the probability of PRRSV RNA detection using RT-qPCR has been demonstrated in different swine sample types (29–31).

Weaning-age pigs are frequently translocated and thus play a role in the spread of swine disease pathogens (32); in addition, many studies have demonstrated that surveiling this subpopulation this subpopulation is more effective in accurately determining the shedding status of breeding herds (33, 34). The American Association of Swine Veterinarians (AASV) PRRSV classification scheme for breeding herds recognizes serum samples from weaning-age pigs as the most suitable sample for assessing the PRRSV shedding status of PRRSV-positive herds (35). In the recently revised version of the mentioned classification scheme, the family oral fluid (FOF) sample is the only other sample type obtainable from this subpopulation of pigs that can be used as supporting evidence for breeding herds to stay within or move between categories.

The primary objective of this study was to compare the PRRSV RNA detection rates in serum, oral swabs (OS), nasal swabs (NS), and ear-vein blood swabs (ES) obtained under field conditions from the weaning-age pigs naturally exposed to wild-type PRRSV. A secondary objective of this study was to evaluate the RT-qPCR detection of PRRSV RNA in FOF samples and swab pools (*comprising individual swab samples from all the piglets within a litter*).

## 2. Materials and methods

### 2.1. Study type

This cross-sectional field study was conducted in three commercial breeding herds from three production systems naturally exposed to wild-type PRRSV-2. This study was approved by the Institutional Animal Care and Use Committee (IACUC) of Iowa State University, IA, USA, under protocol number IACUC-22-101.

### 2.2. Eligibility criteria

PRRSV-positive unstable breeding herds in the Midwestern U.S. were conveniently selected; these herds had low (*n* = 1) to high (*n* = 2) PRRSV prevalence as defined by the modified AASV PRRSV classification of breeding herds (36). PRRSV-unstable low prevalence (AASV status 1B) breeding herds had at least 10 of 13 consecutive weekly PRRSV-negative RT-qPCR tests on processing fluids, while PRRSV-unstable high prevalence (AASV 1A) had <10 of 13 negative tests. These herds did not have piglets vaccinated at processing or weaning (until sampling for the study was completed).

### 2.3. Sample size justification and sampling

A sample size of 620 piglets provided an 80% power to detect a difference in PRRSV detection rates between serum and any of the swab sample types with 95% confidence, assuming that the probability of RT-qPCR tests on serum to correctly identify a PRRSV-positive animal was 95%, the probability of RT-qPCR tests on any swab sample to correctly identify a PRRSV-positive animal was 80%, and PRRSV was at ≥10% prevalence in the sampled population (37–39).

To obtain this sample size, the study was designed to sample 20 weaning-age (18 to 21 days of age) litters each from three eligible herds. With an estimated mean litter size of 11 piglets, the projected number of piglets to be sampled across all herds became 660.

Serum, OS, NS, and ES were collected from individual piglets. FOF samples were collected from each litter. At eligible farms, farrowing rooms to be sampled were selected based on an earlier RT-qPCR-positive test on processing fluids.

### 2.4. Sample collection

Blood samples were collected via jugular venipuncture using single-use serum separation vacutainer tubes (B.D. Vacutainer^®^, Becton Dickinson and Company, Franklin Lakes, NJ, USA) on physically restrained piglets. After blood coagulation, serum was decanted into 5 ml falcon tubes using Pasteur pipettes.

ES were obtained from physically retrained pigs; for most pigs, the ear vein was visible enough to be pricked. However, in a few cases, manual pressure was applied to the base of one ear to enhance the visibility of the lateral auricular vein; a $20G\left(0\;.90mm\right)\;x\;1\frac{1}{2}$" (38mm) needle (Exelint International Co., Redondo Beach, CA., USA) was thereafter used to prick the vein, and a 6” Puritan^®^ sterile polyester-tipped applicator (Puritan Medical Products Company, LLC, Guilford, ME, USA) was used to collect blood seeping from the venipuncture. The applicator sticks were thereafter transferred to an appropriately labeled tube containing 1X phosphate-buffered saline (PBS) sterile solution (RPI Research Products International, Mt. Prospect, IL, USA).

OS and NS samples were also collected similarly from physically restrained pigs using a 6” Puritan^®^ sterile polyester-tipped applicator (Puritan Medical Products Company LLC, Guilford, ME, USA). For OS samples, the swab stick was rotated a few times as far back into the mouth as possible, while NS samples required the applicator to be rotated deep into both nostrils with minimal force. After collection, the swab was transferred to an appropriately labeled tube containing 2 ml of PBS, as previously described.

FOF samples were collected from litters using an untreated single-cord cotton rope tied to the side rail of each sampled farrowing crate to reach about the shoulder level of the piglets. The sow and piglets were allowed to interact with the ropes for approximately 10 to 30 min. Fluids were then wrung off the chewed ropes into 50-ml Falcon tubes (Corning Science Mexico S.A. de C.V., Tamaulipas, Mexico).

All matched samples were stored on ice and transported to the Iowa State University Veterinary Diagnostic Laboratory for RT-qPCR testing using previously validated protocols, quality assurance, and quality control procedures in place.

### 2.5. RT-qPCR testing of samples

#### 2.5.1. Sample preparation and plating

Swab samples were vortexed for 5 seconds, and 250 μl from each swab sample was transferred into dummy plates (Costar^®^ Assay Plate, Corning Inc., Corning, NY, USA).

Each swab sample type (ES, NS, and OS) was also pooled by litter; 250 μl of each swab sample was transferred to an appropriately labeled 5 ml Falcon tube and vortexed for 10 s at 3,200 revolutions per minute. Afterward, 250 μl of the resulting pool was transferred to a dummy plate. The dummy plates were then stored for up to 2 h at 4°C before proper plating for extraction. All samples were tested at the Iowa State University College of Veterinary Medicine Research and Development Laboratory for PRRSV-2 RNA by RT-qPCR to confirm PRRSV status and establish Ct values.

#### 2.5.2. PRRSV RNA Extraction and RT-qPCR

Nucleic acids were extracted from all study samples using the same commercial kit (RealPCR^*^ DNA/RNA Magnetic Bead Kit, IDEXX Laboratories, Inc., One IDEXX Drive, Westbrook, ME, USA) and automated extraction equipment (Kingfisher Flex System Magnetic Beads Processor, Thermo-Fisher Scientific, Waltham, Massachusetts, USA) at room temperature. Summarily, 200 μl of sample and 200 μl of lysis buffer solution were incubated together for 15 min and then jointly incubated for 5 min with a prepared magnetic bead solution (600 μl binding buffer + 20 μl magnetic beads). The magnetic beads were then retrieved and washed for 3 min with 600 μl of Wash solution I, 600 μl of Wash solution II, and 600 μl of 80% ethanol (in this order) and then allowed to dry for 10 min. The nucleic acids were thereafter eluted from the magnetic beads using a 100 μl elution buffer solution for 5 min.

PRRSV RT-qPCR was then performed on the extracted nucleic acids using the RealPCR^*^ PRRSV-1 and PRRSV-2 Multiplex RNA Mix and Master Mixes (IDEXX Laboratories, Inc., Westbrook, ME, USA). Briefly, 5 μl of eluted nucleic acids were transferred to PCR plate wells containing 10 μl of the multiplex RNA mix and 10 μl of the Master mix (summing up to 25 μl per well). These plates were then loaded onto a thermal cycler (7500 Fast Real-Time PCR System, Applied Biosystems, Foster City, California, USA), and the following cycling conditions with a standard ramp rate were used: (1) one reverse transcription cycle at a temperature of 50°C for 15 min, (2) one denaturation cycle at a temperature of 95°C for 1 min, and (3) 45 amplification cycles, each having a set temperature of 95°C for 15 s and 60°C for 30 s. Amplification data were analyzed using the ‘auto baseline' with the Cycle threshold manually adjusted to 10% of the peak reading of the positive amplification control (IDEXX Laboratories, Inc., Westbrook, ME, USA). According to the manufacturer's recommendations, samples with Ct values <40 were considered PRRSV-positive.

Each RT-qPCR run included assay controls comprising a negative extraction control (a known PRRSV-negative pig sample), a positive extraction control (a known PRRSV-positive pig sample), a negative amplification control (nuclease-free water), and a positive amplification control (IDEXX Laboratories, Inc., Westbrook, ME, USA).

### 2.6. Statistical analyses and modeling

#### 2.6.1. Overview

Tables and plots were used to describe relevant attributes of the sampled farms, samples obtained, samples tested, and RT-qPCR results. To facilitate analyses, the study samples were further re-categorized into two groups:

A. Piglet-level sample types: These are individual piglet samples and include OS, NS, ES, and serum.
B. Litter-level sample types: These are aggregated or pooled samples that each represent an entire litter of piglets and include FOF, OS pools, NS pools, and ES pools.

For the piglet-level samples, plots were used to illustrate the relationship between OS, NS, and ES Ct values and serum Ct values. The relationship between the proportion of viremic piglets within a litter (serum within-litter prevalence) and the proportion of piglets with RT-qPCR positive swabs within litters (swab within-litter prevalence) was also illustrated. Logistic regression models were built to assess the probability of PRRSV detection in a swab sample, given the level of viremia (serum RT-qPCR Ct) in the piglets.

For the litter-level samples, plots were used to illustrate PRRSV detection in swab pools with changes in the proportion of viremic piglets within the litter. Models were also used to characterize the probability of PRRSV detection in the litter-level samples, given the proportion of- and Ct values of component swab samples within the pools.

Two-by-two contingency tables were also constructed to assess agreement between piglet and litter-level sample types. The diagnostic performance of the swabs at the piglet-level, and all litter-level sample types.

#### 2.6.2. Farms and samples

The location, estimated prevalence, circulating PRRSV-2 variant, number of litters sampled, and within-litter prevalence of the sampled farms are illustrated in figures and tables. The figures were built using the ggplot2 package on R statistical software (40). The results from RT-qPCR tests conducted on serum samples were considered to represent the true PRRSV status of the sampled piglets; hence, the serum was the reference sample for estimating within-litter prevalence and determining a truly positive piglet or litter.

For each of the three sampled herds, farm prevalence $\left(P_{j}^{Farm}\right)$ was calculated as the proportion of sampled piglets that were PRRSV RT-qPCR-positive using sample type *j* (*where j* = *OS, NS, ES or Serum*).


(1) $$\begin{array}{l} P_{j}^{Farm}\;=\frac{number\;of\;RTqPCR\;positive\;piglets\;}{Total\;number\;of\;piglets\;sampled\;from\;farm} \end{array}$$

For each sampled litter, the within-litter prevalence $\left(P_{j}^{Litter}\right)$ was calculated as the proportion of piglets that were PRRSV RT-qPCR-positive using sample type *j* (*where j* = *OS, NS, ES or Serum*).


(2) $$\begin{array}{l} P_{j}^{Litter}\;=\frac{number\;of\;RTqPCR\;positive\;piglets\;within\;litter\;}{Total\;number\;of\;piglets\;within\;litter} \end{array}$$

##### 2.6.2.1. Piglet-level samples

Boxplots were used to assess the distribution of Ct-values from RT-qPCR testing on serum, OS, NS, and ES samples. Scatter plots were also used to assess the relationship between the serum Ct values and the Ct values of each swab sample type. The linear relationship between the serum Ct values and the Ct values of each swab sample type was assessed using the Pearson correlation coefficient (R) (41), and the accompanying *p*-value was obtained; both obtained values were and embedded on the scatterplots mentioned above using the *ggmisc* package (42) on R statistical software (40).

Scatter (X.Y) plots were also used to assess the relationship between $P_{Srm}^{Litter}\left(predictor\;variable\right)$ and $P_{OS}^{Litter}$, $P_{NS}^{Litter}$, and $P_{ES}^{Litter}\left(response\;variables\right)$. Colored shapes were used to indicate ranges of the mean Ct of viremic piglets within a litter. For this study, a viremic piglet was defined as any piglet with detectable quantities of PRRSV RNA in its serum sample (in other words, having a serum Ct value <40). Mathematically, the mean Ct (MCt) using the sample type *j* (*where j* = *OS, NS, ES or Serum*) can be expressed as follows:


(3) $$MCt_{j}=\frac{\sum_{i=1}^{n_{p}}\left(Ct_{p}\right)}{n_{p}}\;,$$

where *Ct*_*p*_ is the Ct value for each PRRSV-positive piglet *i* within a litter, and *n*_*p*_ is the number of RT-qPCR-positive piglets within that litter using sample type *j*.

Separate generalized linear models were used to estimate the probability of PRRSV detection in each of the alternative sample types given the Ct value of serum. For


 i=1,…, 96

let *Y*_*i*_ be the response variable associated with PRRSV detection in the swab (ES, NS, or OS) of the *i*^*th*^ viremic piglet, and *Y*∈(0, 1).

Let *X*_*i*_ be the predictor variable associated with the RT- qPCR Ct of serum from the *i*^*th*^ viremic piglet, and logit $\left(Y_{i}\right)=\beta_{0}+\;{\beta_{1}^{*}X}_{i}$.

The estimated probability of detection for each swab sample type is calculated as follows:


(4) $$\begin{array}{l} \frac{e^{\beta_{0}+{\beta_{1}^{*}X}_{i}}}{1+\;e^{\beta_{0}+{\beta_{1}^{*}X}_{i}}}, \end{array}$$

where *β*_0_ *is* the model's intercept, and *β*_1_is the model's regression coefficient.

Two-by-two contingency tables were built to assess the tests' agreement [crude agreement and Cohen's kappa (43)] and evaluate the diagnostic performance of each of the swab sample types. To evaluate the diagnostic performance (sensitivity and specificity) of the swab sample types, a piglet was considered truly positive if it was viremic. The formulas used for calculating the above are well described (Supplementary Table S9, Text S1) and the scale (43) for the interpretation of the Kappa statistic is given in Supplementary Table S7.

##### 2.6.2.2. Litter-level samples

Boxplots were used to assess the distribution of Ct-values from RT-qPCR tests conducted on OSp, NSp, ESp, and FOF samples. Separate generalized linear models were used to estimate the probability of PRRSV detection in a swab pool for each swab sample type, given the proportion of positive samples within the pools.

Two-by-two contingency tables were also built to assess the tests' agreement and estimate the diagnostic performance of each litter-level sample type. To evaluate the diagnostic performance of the litter-level sample types, the reference status of each litter was the presence or absence of at least one viremic piglet; in other words, a litter was considered truly positive if it had at least one PRRSV RT-qPCR-positive test on piglet serum.

## 3. Results

### 3.1. Farms and samples

A total of 666 piglets were sampled, representing 55 litters across all three farms. The locations of the farms, time from the last outbreak (as reported by the attending veterinarian) to sampling, AASV PRRSV status, restriction fragment length polymorphism (RFLP) of the open reading frame- 5 gene, PRRSV genetic lineage, the number of litters sampled, the number of piglets sampled, and the number of crates in the sampled room(s) are presented in Table 1. The total number of test samples by farm is summarized in Supplementary Table S8; in total, 2,882 test samples were analyzed across all three farms.

**Table 1:** Summary of farm characteristics for location, time from outbreak to sampling, herd PRRSV status, ORF-5 RFLP and lineage, and room characteristics.

**Farm (location)**	**Time from outbreak to sampling**	**Herd status**	**ORF-5 RFLP and Lineage**	**Litters/crates sampled (piglets)**	**Room characteristics**
Farm 1 (Muscatine, Iowa)	7 months	Low PRRSV-prevalence	1-4-4 1A	20 (229)	Two rooms with 24 crates each
Farm 2 (Washington, Iowa)	3 months	High PRRSV-prevalence	1-8-4 1H	15 (192)	One room with 64 crates
Farm 3 (Fairbury, Nebraska)	9 months	High PRRSV-prevalence	1-8-4 1H	20 (245)	One room with 56 crates

### 3.2. Sampling

**Figure 1:**
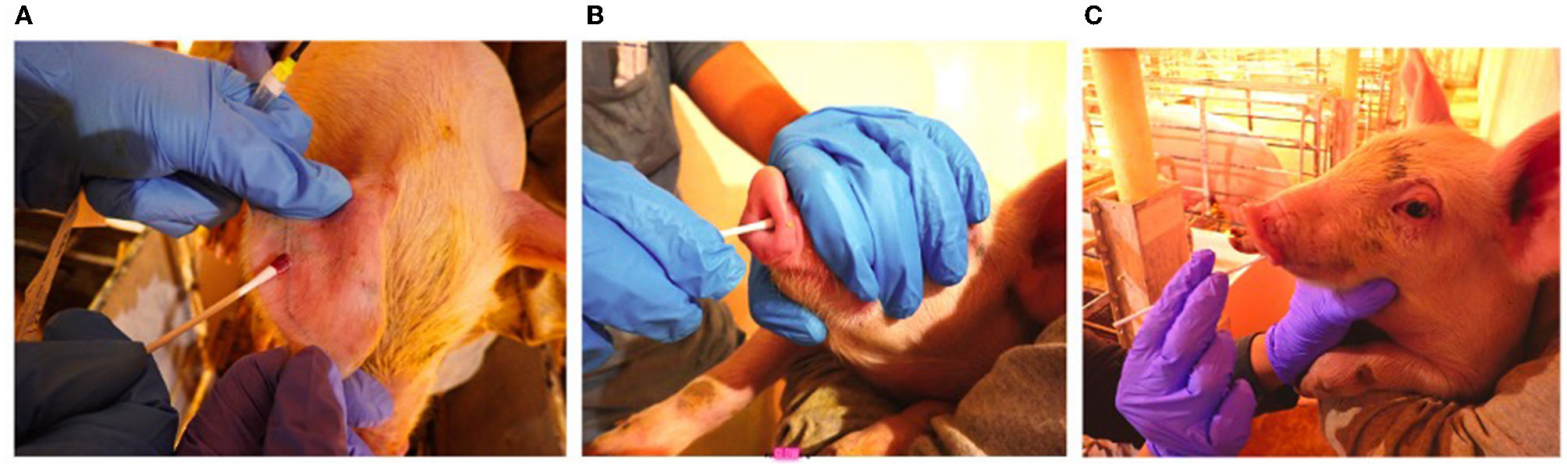
Swab collection **(A)** Ear-vein blood swab, **(B)** Nasal swab, **(C)** Oral swab.

Pictorial illustrations of swab collection are shown in Figure 1.

**Figure 2:**
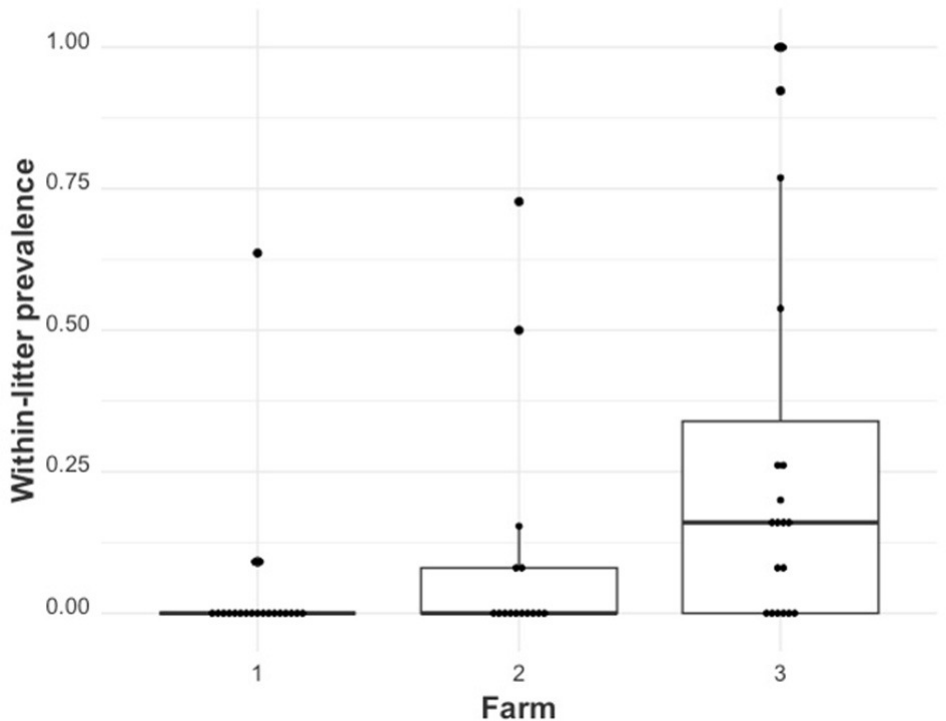
The distribution of within-litter PRRSV prevalence ($P_{serum}^{Litter}$) by farm (farms 1, 2, and 3).

The distribution of within-litter prevalence across farms is shown in Figure 2. Farm 1 had 9 (3.9%) RT-qPCR-positive piglets distributed across 3 of the 20 sampled litters. Farm 2 had 18 (9.4%) RT-qPCR-positive piglets distributed across 5 of the 15 sampled litters. Farm 3 had 69 (28.16%) RT-qPCR-positive piglets distributed across 14 of the 20 sampled litters. The maximum within-litter prevalence values were 0.64, 0.73, and 100% for Farms 1, 2, and 3, respectively.

### 3.3. Piglet-level samples

#### 3.3.1. General description

**Figure 3:**
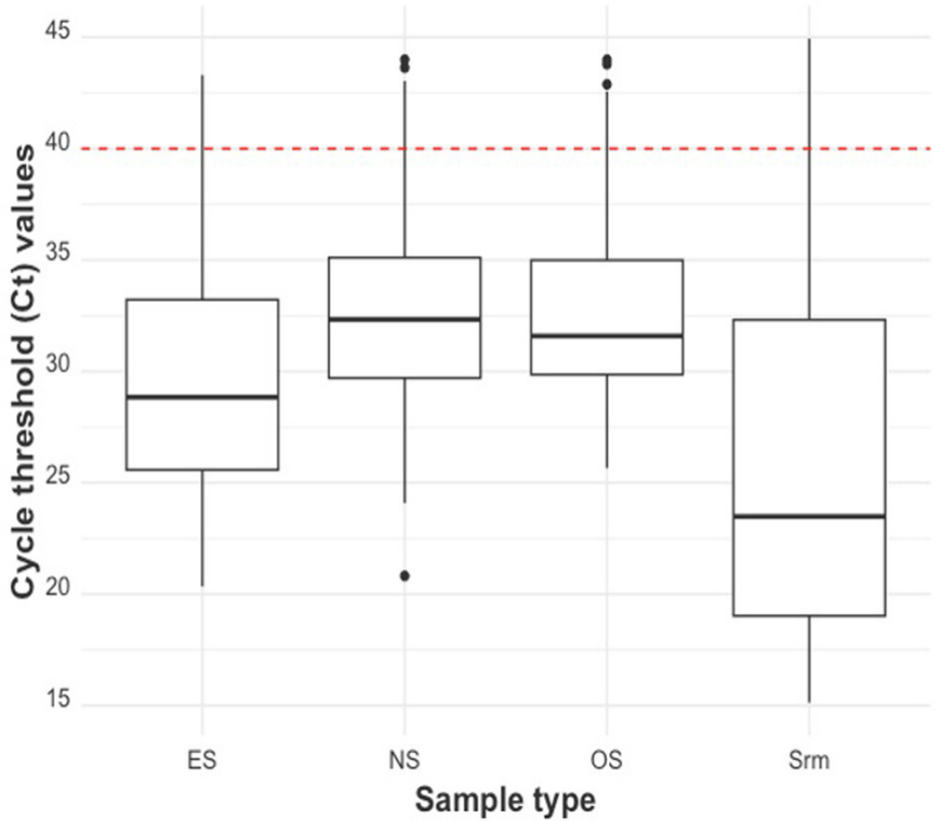
Ct distribution by piglet-level sample type (from left to right: ear-vein blood swabs, nasal swabs, oral swabs, and serum). The red dashed line indicates the cut-off Ct value for categorizing a sample as positive (<40) or negative (≥40).

The distribution of the Ct values of the piglet-level sample types was shown using boxplots (Figure 3). Serum had the lowest median Ct at 23.48 (21.65 if only Ct values <40 were considered), followed by ES (28.84; 28.77 if only Ct values <40 were considered), and OS (31.59; 31.14 if only Ct values <40 were considered). NS had the highest median Ct value at 32.33 (31.94 if only Ct values <40 were considered).

**Figure 4:**
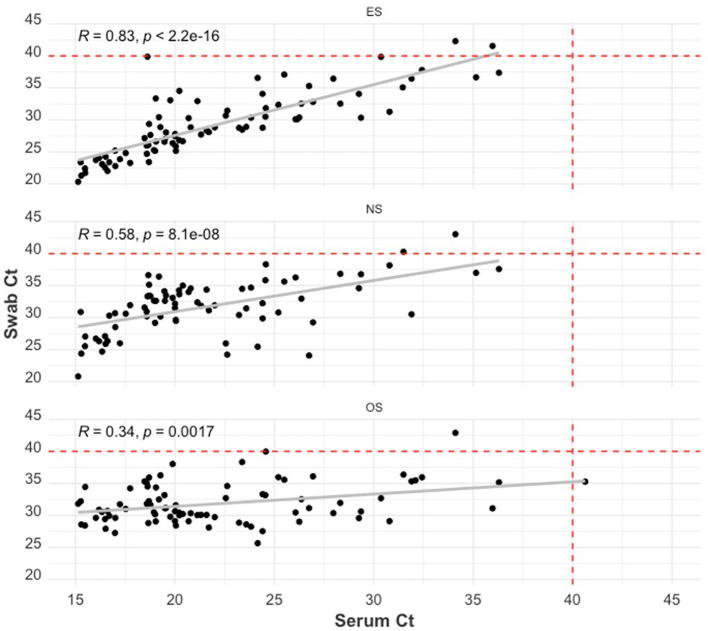
Scatter plot of the RT-qPCR Ct of the swab sample types: ear-vein blood swabs **(top)**, nasal swabs **(middle)**, and oral swabs **(bottom)** to the RT-qPCR Ct of serum samples. The red dashed lines indicate the cut-off Ct value for categorizing a sample as positive (<40) or negative (≥40). The Pearson correlation coefficient (R) and the *p*-value of this coefficient are shown on each plot.

The relationship between swab Ct and serum Ct is illustrated using scatterplots in Figure 4. All swab sample Ct values were positively correlated with serum Ct values (*p*−*values* < 0.01). ES Ct and serum Ct had the highest Pearson correlation coefficient value (R) of 0.83, while the OS Ct by serum Ct comparison had the lowest correlation (R = 0.34).

**Figure 5:**
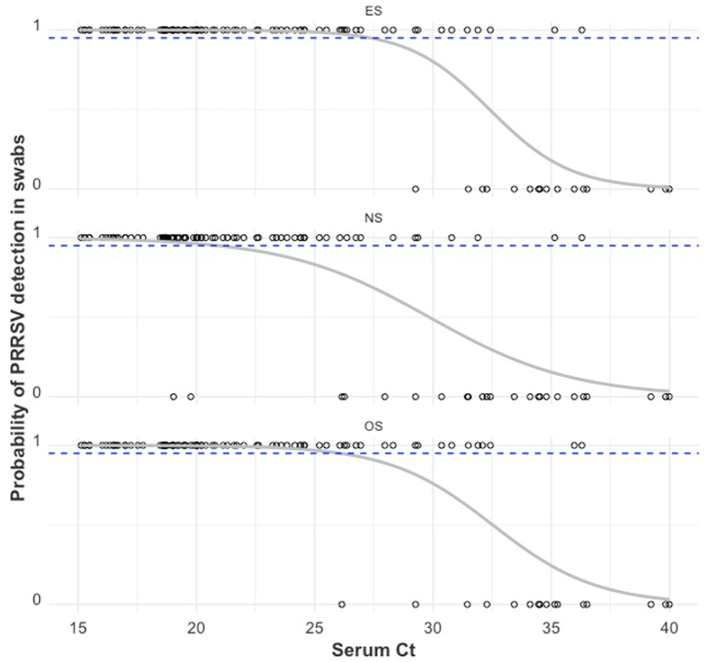
PRRSV RNA detection in a swab sample (1 = positive sample with Ct < 40, 0 = negative sample with Ct ≥ 40) by the RT-qPCR Ct of serum obtained from that pig. The gray curve is the estimated probability of detection. The dashed blue line indicates the estimated 95% probability of detection.

A plot of PRRSV RNA detection in a swab sample compared to the serum Ct of that piglet is shown in Figure 5. For all swab sample types, the lower the serum Ct of a piglet, the higher the probability of detection of PRRSV RNA in the swab. There was a ≥95% probability of detection in ES, OS, and NS when the serum Ct in the piglet was ≤ 27.43, ≤ 25.48, and ≤ 21.05, respectively. As can be observed from Table 2, when the serum Ct was ≤ 25, the ES and OS were always positive (61/61), while the NS were almost always positive (59/61). For serum Ct between 25 and 35, OS and ES had similar positivity rates (17/26), while NS had a lower positivity rate (11/26). For Serum Ct between 35 and 40, all three swab samples had similar positivity rates (2/9).

**Table 2:** Description of RT-qPCR positive serum Ct ranges, total number of piglets within those ranges, and the percentage and number of positive samples for ear-vein blood swabs, nasal swabs, and oral swabs within each range.

**Serum Ct range**	**Number of piglets**	**Positive ES swabs**		**Positive NS swab**		**Positive OS swabs**	
		**%**	**Number**	**%**	**Number**	**%**	**Number**
15–20	36	100	36	94.4	34	100	36
20–25	25	100	25	100.0	25	100	25
25–30	13	92.3	12	69.2	9	84.6	11
30–35	13	38.5	5	15.4	2	46.2	6
35–40	9	22.2	2	22.2	2	22.2	2

**Figure 6:**
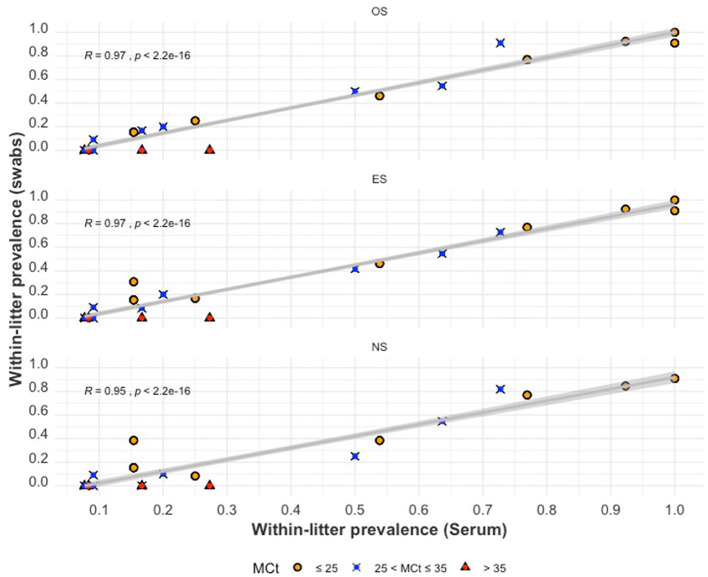
The within-litter PRRSV prevalence for oral swabs **(top)**, ear-vein blood swabs **(middle)**, and nasal swabs **(bottom)** compared to serum within-litter prevalence. The colored shapes indicate the mean Ct values (*MCt*_*serum*_) of positive serum samples from a litter.

The relationship between within-litter prevalence by swabs ($P_{ES}^{Litter},P_{NS}^{Litter},{and\;P}_{OS}^{Litter}$) and within-litter prevalence by serum ($P_{Srm}^{Litter}$) is illustrated using scatterplots (Figure 6). Positive correlations were found for $P_{ES}^{Litter},P_{NS}^{Litter},{and\;P}_{OS}^{Litter}$ with $P_{Srm}^{Litter}$ (*P*-value < 0.001). The highest Pearson correlation coefficient value (R) was observed for $P_{ES}^{Litter}$ and $P_{OS}^{Litter}$ (*R* = 0.97), while $P_{NS}^{Litter}$ had a slightly lesser correlation (R = 0.95). When the mean Ct of serum samples (*MCt*_*serum*_) was >35, the within-litter prevalence for all swabs was zero (no piglet within those litters tested positive using swab samples). The relationship between $P_{Srm}^{Litter}$ and each of $P_{ES}^{Litter},P_{NS}^{Litter},{and\;P}_{OS}^{Litter}$appears to be more linear as $P_{Srm}^{Litter}$ increases.

#### 3.3.2. Agreements and diagnostic performance

The raw results of the two-by-two comparisons of all paired combinations of the piglet-level sample types are summarized in Supplementary Tables S1, S2. The crude agreement and Cohen's kappa values for all paired combinations of the piglet-level sample types are summarized in Supplementary Table S3.

There was ≥95% agreement, and *C*_*k*_ ≥ 0.81 across all sample-type comparisons, with the highest crude agreement (0.98) and *C*_*k*_ (0.91) being between ES and NS, and the least agreement between serum and NS (0.96 crude agreement and 0.81 *C*_*k*_).

The diagnostic performances of the swab sample types are summarized in Table 3. ES had the highest sensitivity (0.83) and specificity (≈1.00) of all three swabs. OS was as sensitive as ES, and NS was the least sensitive (0.75). All swab samples had relatively high specificity (≈ ≥0.99).

**Table 3:** The diagnostic evaluation (and 95% confidence intervals) of the RT-qPCR detection of PRRSV in weaning-age pigs using ear-vein blood swabs (ES), nasal swabs (NS), and oral swabs (OS) with serum samples as the reference sample.

** 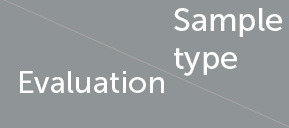 **			
	**ES**	**NS**	**OS**
Crude agreement	0.97	0.96	0.97
Cohen's kappa	0.88 (0.83, 0.94)	0.81 (0.75, 0.88)	0.85 (0.80, 0.91)
Sensitivity	0.83 (0.74, 0.90)	0.75 (0.65, 0.83)	0.83 (0.74, 0.90)
Specificity	1.00 (0.99, 1.00)	0.99 (0.98, 1.00)	0.99 (0.97, 1.00)
Positive predictive value	0.98 (0.91, 1.00)	0.96 (0.94, 0.97)	0.92 (0.84, 0.97)
Negative predictive value	0.97 (0.96, 0.98)	0.95 (0.87, 0.99)	0.97 (0.96, 0.98)

Using the *epi.ssdetect* function within the epiR package (44) on R statistical software, the RT-qPCR sensitivity and specificity estimates for each piglet-level sample were used to calculate appropriate sample sizes for detecting at least one positive pig with 95% confidence in a population of ≥1,000 pigs (Table 4).

**Table 4:** Sample size estimates for serum, ear-vein blood swabs, nasal swabs, and oral swabs using the sensitivity and specificity estimates from this study, a 95% confidence, a perfect RT-qPCR test, and sampling without replacement.

** 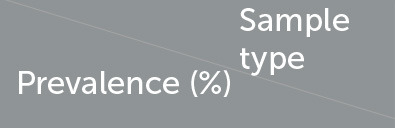 **			
	**Serum**	**ES**	**NS**	**OS**
1	259	312	346	312
3	96	115	127	115
5	59	71	78	71
10	30	36	40	36
20	15	18	20	18
50	6	8	8	8

### 3.4. Litter-level sample-types

#### 3.4.1. General description

**Figure 7:**
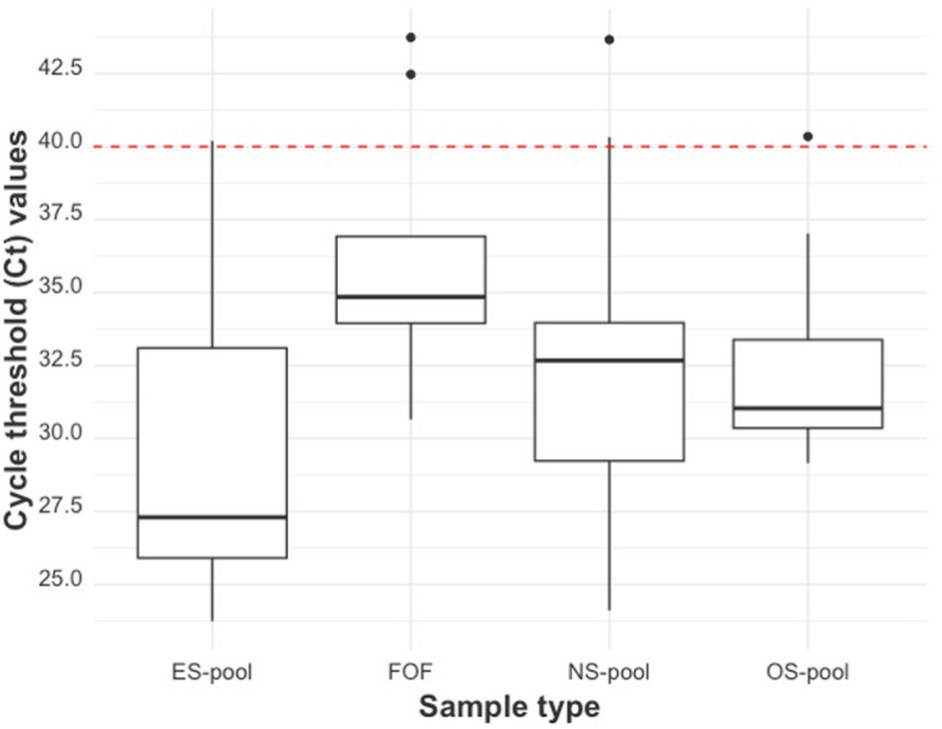
The RT-qPCR Ct distribution by litter-level sample type. From left to right: ear-vein blood swab pools, family oral fluids, nasal swab pools, and oral swab pools. The red dashed line indicates the cut-off Ct value for categorizing a sample as positive (<40) or negative (≥40).

The distribution of Cts of the litter-level sample types is shown using boxplots (Figure 7). ES pools had the lowest median Ct of 27.30 (27.19 if only Ct values <40 were considered), OS pools had the next lowest median Ct value of 31.04 (30.90 if only Ct values <40 were considered), NS pools had the next lowest median Ct value of 32.67 (32.08 if only Ct values <40 were considered), and FOF pools had the highest median Ct value of 34.85 (34.61 if only Ct values <40 were considered). The variations in the mean Cts of the litter-level samples compared to the mean Ct of serum are shown in Supplementary Figure S1.

**Figure 8:**
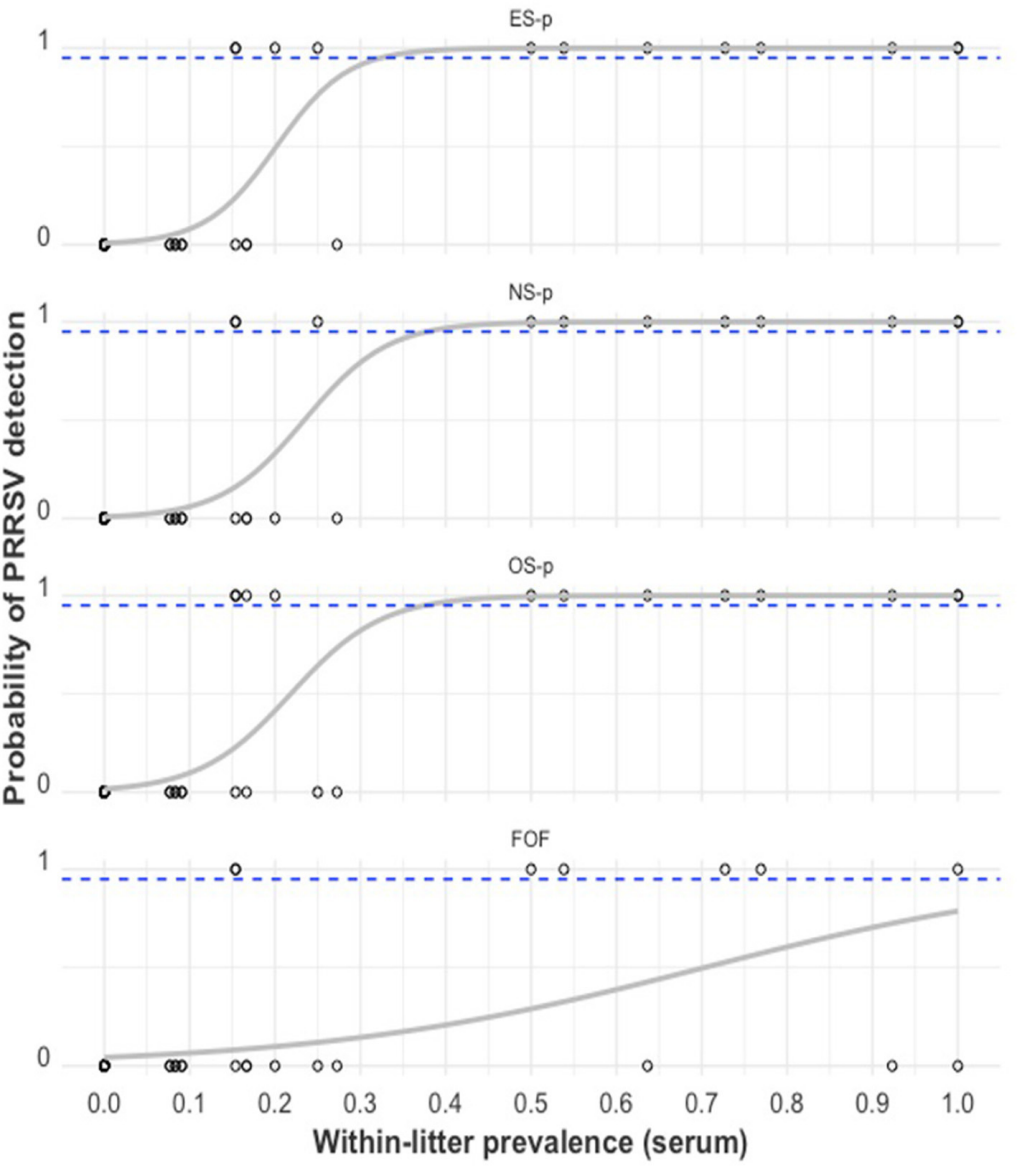
PRRSV RT-qPCR detection in swab-pool samples (1 = positive sample with Ct <40, 0 = negative sample with Ct ≥ 40) by serum within-litter prevalence. The gray curve is the estimated probability of detection. The dashed blue line indicates the estimated 95% probability of detection. From top to bottom: ear-vein blood swab pools (ES-p), nasal swab pools (NS-p), oral swab pools (OS-p), and family oral fluids (FOF).

The relationship between PRRSV detection in a litter-level sample type and the serum within-litter prevalence (or $P_{Srm}^{Litter})\;$is illustrated in Figure 8. As the proportion of viremic piglets within a little increased, the probability of detecting PRRSV through RT-qPCR also increased for all litter-level samples. There is a ≥95% probability of detection in ES-, NS-, and OS pools when the proportion of viremic piglets in the litter was ≥32.4%, 37.8%, and 37.6%, respectively.

**Figure 9:**
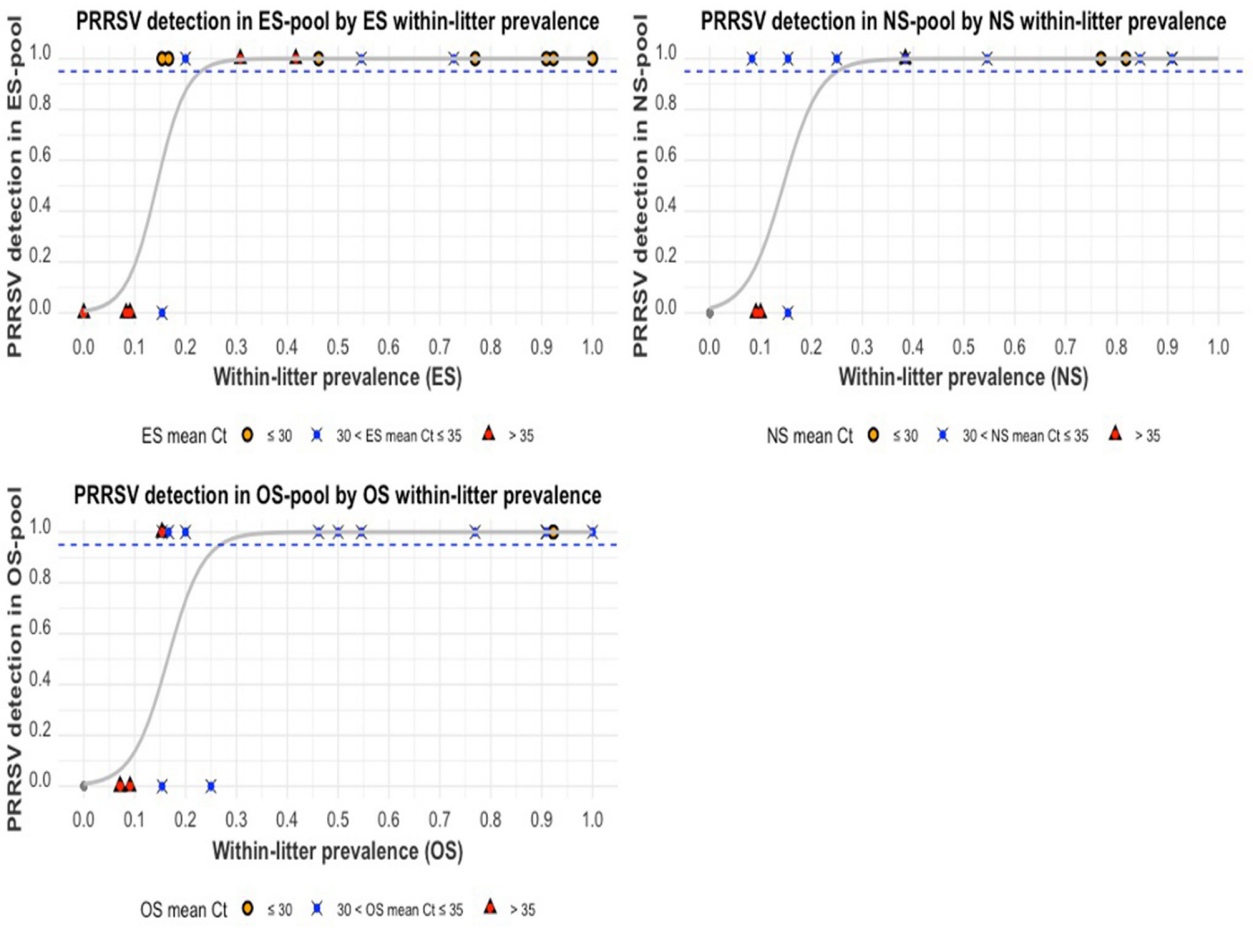
The probability of PRRSV RNA detection in pooled swab samples compared to the proportion of positive swab samples within pools: ear-vein blood swab pools **(top-left)**, nasal swab pools **(top-right)**, and oral swab pools **(bottom-left)**. The gray curve is the estimated probability of detection. The dashed blue line indicates the estimated 95% probability of detection, and the colored shapes indicate the mean Ct values of the positive swab samples within a pool.

An assessment of the probability of PRRSV detection in a swab pool, given the proportion of positive swab samples within that pool, is illustrated in Figure 9. The probability of PRRSV detection in a pool increased with the proportion of positive samples within the pool. There was a lower probability of detection when the mean Ct of positive samples within a pool was >35. When $P_{ES}^{Litter}$ was ≥23%, there is a ≥95% probability of PRRSV detection in an ES pool. When $P_{OS}^{Litter}$ was ≥27%, there is a ≥95% probability of PRRSV detection in an OS pool. When $P_{NS}^{Litter}$ was ≥26%, there is a ≥95% probability of PRRSV detection in an NS pool. Additionally, litters with higher mean Cts (or lower viral loads) had fewer PRRSV-positive piglets within them.

#### 3.4.2. Agreement and diagnostic performance

The raw results of the two-by-two comparisons of all paired combinations of the litter-level sample types are summarized in Supplementary Table S4. The crude agreement and Cohen's kappa values for all paired combinations of the piglet-level sample types are also summarized in Supplementary Table S5.

There was ≥90% agreement, and *C*_*k*_ ≥ 0.68 across all sample-type comparisons, with the highest agreement being between ES pools and NS pools (0.98 agreement and *C*_*k*_ = 0.91) and the least agreement between FOF and ES pools (0.91 agreement and *C*_*k*_ = 0.68).

A comparison of RT-qPCR results of the litter-level samples to the true PRRSV status of litters is shown in Supplementary Table S6. The diagnostic performances of the litter-level sample types are summarized in Table 5. ES pools and OS pools had the highest sensitivity (0.55) of all four sample types. NS pools and FOF had a sensitivity of 0.50 and 0.32, respectively. All litter-level samples had a specificity of 1.00 (100%).

**Table 5:** The diagnostic evaluation (and 95% confidence intervals) of the RT-qPCR detection of PRRSV in weaning-age pigs using family oral fluids (FOF) and litter pools of ear-vein blood swabs (ES), nasal swabs (NS), and oral swabs (OS).

** 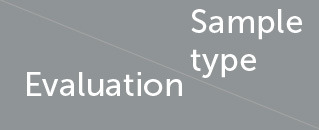 **				
	**ES-pool**	**NS-pool**	**OS-pool**	**FOF**
Crude agreement	0.82	0.80	0.82	0.73
Cohen's kappa	0.59 (0.38, 0.80)	0.55 (0.33, 0.76)	0.59 (0.38, 0.80)	0.36 (0.15, 0.57)
Sensitivity	0.55 (0.32, 0.76)	0.50 (0.28, 0.72)	0.55 (0.32, 0.76)	0.32 (0.14, 0.55)
Specificity	1.00 (0.89, 1.00)	1.00 (0.89, 1.00)	1.00 (0.89, 1.00)	1.00 (0.89, 1.00)
Positive predictive value	1.00 (0.74, 1.00)	1.00 (0.72, 1.00)	1.00 (0.74, 1.00)	1.00 (0.59, 1.00)
Negative predictive value	0.77 (0.61, 0.88)	0.75 (0.60, 0.87)	0.77 (0.61, 0.88)	0.69 (0.54, 0.81)

The reference standard for this diagnostic evaluation is the presence of ≥1 viremic piglets within a litter.

## 4. Discussion

The primary goal of this study was to compare the detection of PRRSV RNA through RT-qPCR in swab samples vs. serum samples collected from weaning-age pigs. This goal was achieved, and the effect of sample type on the outcome of PRRSV RT-qPCR tests in weaning-age pigs was demonstrated. This study also demonstrated the effect of litter-level pooling of swab samples on the probability of PRRSV detection using RT-qPCR. The findings of this study are valid for herds sharing similar characteristics as the study herds, and are credible since the appropriate sample size determined *a priori* was achieved (≥620 pigs), and samples were obtained under practical field conditions from multiple herds naturally exposed to wild-type PRRSV.

The heterogeneity in the location, PRRSV prevalence, circulating PRRSV variant, and management (different production systems) of the sampled herds was aimed at strengthening the validity of the diagnostic accuracy estimates. This study does not attempt to account for every variable that could have influenced PRRSV RNA detection dynamics in weaning-age pig populations but was rather, focused on utilizing cross-sectional “snapshots” to reasonably demonstrate how PRRSV RT-qPCR detection rates could vary across sample types in weaning-age pig populations.

Nasal swabs have demonstrated use in the surveillance of various porcine respiratory pathogens, including *Mycoplasma hyopneumoniae* (45, 46), porcine circoviruses (47–50), parainfluenza virus (51, 52), influenza A virus (24, 25, 53), *Pasteurella multocida* (54, 55), *Actinobacillus pleuropneumoniae* (56), and PRRSV (18). The use of oral swabs in surveilling swine respiratory pathogens has not gained as much traction as its more passively obtained counterpart, the oral fluid sample (57). Nonetheless, oral swab use has been demonstrated in older pig populations (58, 59) and, more recently, weaning-age pigs (23). The effectiveness of using ear-vein blood swabs for PRRSV surveillance has been demonstrated in boars (60). There have also been reports elsewhere of the use of ear-vein blood swabs for PRRSV-1 surveillance (16). To the best of our knowledge, this is the first study that has evaluated PRRSV RNA detection in ear-vein blood swabs, nasal swabs, and their pools specifically for naturally infected weaning-age pigs under field conditions. Swine practitioners can use the provided estimates of specificity and sensitivity to ascertain appropriate sample sizes for surveilling PRRSV using swab samples in weaning-age pigs. For example, given the sensitivity and specificity estimates provided in Table 3, a minimum number of 30, 36, and 40 piglets need to be sampled via serum, ES, OS, and NS, respectively, to achieve a 95% confidence in detecting ≥1 PRRSV-positive piglet when the PRRSV prevalence is at least 10% (Table 4).

For diagnostic accuracy studies where the disease status of each sampled animal is neither known nor established *a priori*, Cohen's kappa statistic is a more appropriate tool than the McNemar chi-square test in evaluating two-by-two tables (61–63). While Cohen's kappa test assesses the agreement beyond chance using all four quadrants of the two-by-two tables, the McNemar test uses the discordant pair in the two-by-two tables to assess bias in the “new” test compared to the reference or gold standard. Neither of the tests evaluates sensitivity or specificity directly. The Cohen's kappa values shown in Supplementary Table S3 demonstrate near-perfect agreement (63) (Supplementary Table S7) between RT-qPCR tests on all pairs of the piglet-level sample types. Regarding the diagnostic performance of the swab samples, OS and ES PRRSV RT-qPCR tests correctly identified 83% of viremic piglets, while similar tests on NS identified 75% of such animals. While it is not uncommon or rare to find PRRSV RNA in non-viremic pigs' mouths or nasal cavities, detecting PRRSV RNA in the ES of two non-viremic piglets was unexpected. In one of these piglets, no other sample type tested positive, whereas, in the second piglet, the nasal swab also tested positive. Considering the numerous reasons (virus-, piglet-, sample-, sampler-, test-, and operator level factors) that could have been responsible for this observation, it is difficult to speculate exactly why; however, ES samples expectedly had the highest specificity (99.65%), as it correctly identified 567 of the 569 non-viremic piglets (Supplementary Tables S2, S5).

The sensitivity and specificity estimates of the swab samples presented in this study are not absolute but are made with reference to serum samples (Table 3). Although serum is the reference sample for ascertaining the true PRRSV status of a pig, the authors are unaware of a study that gives exact numerical values to the diagnostic accuracy of PRRSV RT-qPCR tests on the serum sample itself; nonetheless, it is a fact that PRRSV can indeed be present within the tissues of a pig and not be detectable in serum (64–69).

ES samples were the most diagnostically accurate among the swab samples and were closest in performance to serum samples. In the sampling of ES (and all swab samples), there were subtle variations in the amount of blood (or fluids) obtained from the pigs during sampling; these variations could be pig- or sampler-dependent. Despite this sample-to-sample variation, it was interesting to discover a linear correlation between the Ct values of ES samples and the Ct values of serum samples; this similarity in RT-qPCR detection rates agrees with the study of Gerber et al. (15), which saw the greatest similarity between serum samples and ES samples among all the study samples tested.

The American Association of Swine Veterinarians (AASV) classification scheme for breeding herds (35, 36) considers the RT-qPCR detection of PRRSV RNA in the serum of weaning-age pigs as an appropriate (and preferred) method for assessing the PRRSV shedding status of a breeding herd (35, 36). This would implicitly mean that this classification scheme presupposes that the presence of a viremic weaning-age pig (or a weaning-age pig with detectable quantities of PRRSV RNA in serum) in a herd is sufficient evidence of active PRRSV shedding in that herd at the time of sampling.

From the study results, the relationship between viremia and shedding can be better appreciated in Figure 4 and Table 2. The lower the serum Ct value (or the higher the PRRSV RNA within the serum sample), the higher the probability that a sampled piglet will have detectable quantities of PRRSV RNA in swab samples. A high Ct (relatively low viremia) on serum could mean that the sampled animal was very recently infected or may be at the tail end of the viremic phase, in which case there would be an expectedly minimal chance of PRRSV RNA detection (or shedding) in the nasal mucosa (18, 19) and the buccal mucosa (59). It also follows that the chances of PRRSV RNA detection in the blood swab will significantly decrease due to the dilution effect.

The observed strong association between the level of viremia in a piglet and the probability of PRRSV RT-qPCR detection in swabs is consistent with and further affirms the earlier highlighted presupposition of the AASV breeding herd classification scheme.

Cohen's kappa evaluation of the litter level samples showed substantial to near-perfect (43) agreement between all pairs of sample types. The presence of a good number of low-prevalence litters in this study could explain the poor sensitivities of the swab pools (Table 5). Unlike conventional pooling study designs where analyte detection is assessed across graded levels of dilution, for this study, the field-observed number of- and PRRSV statuses of piglets within a litter naturally determined the success of the RT-qPCR tests in pools. An observation that stood out in the referenced table (Table 5) was the perfect specificity of all litter-level sample types; even though pooling by litter could have negatively affected sensitivity, it improved the specificity of the swab sample types. This finding agrees with another study (70) that observed improved ELISA specificity after samples were pooled.

The relationship between the probability of PRRSV RT-qPCR detection in swab pools and the serum within-litter prevalence or $P_{Serum}^{Litter}$ (Figure 8) is an evaluation of the diagnostic accuracy of the litter-level samples; this relationship demonstrates the effect of the viremic (reference) status of litters on the probability of having a positive RT-qPCR test on a swab pool. However, an assessment of the probability of PRRSV RT-qPCR detection in swab pools compared to the respective within-litter prevalence ($P_{ES}^{Litter},P_{NS}^{Litter},and\;P_{OS}^{Litter}$) of the swab sample types (Figure 9) highlights the effects of specific attributes of component swab samples on the RT-qPCR detection of PRRSV RNA in those pools. The proportion and the mean Ct of PRRSV-RT-qPCR positive samples in a pool (or litter) were clearly shown to be key determinants of PRRSV RT-qPCR detection in swab pools from truly positive litters; the effect of *MCt*_*serum*_ becomes increasingly evident in litters with relatively few PRRSV-positive pigs, in which case the lower mean Ct (higher PRRSV RNA) litters were more likely to produce RT-qPCR-positive pools (Figure 9). ES pools generally had the lowest range of Cts and the best diagnostic performance compared to the other litter-level sample types. The decrease in the probability of PRRSV RT-qPCR detection rates with increasing proportion of PRRSV negative samples within swab pools and increasing Ct of component-positive samples within pools is consistent with previous studies that have evaluated the effect of pooling samples on PRRSV RNA detection by RT-qPCR (29–31, 71, 72).

In the plot comparing $P_{ES}^{Litter}$, $P_{NS}^{Litter},$ and $P_{OS}^{Litter}$ to $P_{Serum}^{Litter}$ (Figure 6), it can be observed that when $P_{Serum}^{Litter}$ was relatively low, there was more variability and less linearity in its relationship with $P_{ES}^{Litter}$, $P_{NS}^{Litter},$ and $P_{OS}^{Litter}$. This observation may be because in some of the low $P_{Serum}^{Litter}$ litters, the few infected (viremic) piglets are index cases that may not have been shedding (OS and NS) detectable quantities of PRRSV RNA at the time of sampling. The observed higher *MCt*_*serum*_ in such litters further supports this hypothesis. As the $P_{Serum}^{Litter}$ increased, going beyond 50%, it can be observed that there was an almost perfect linear relationship between the proportion of pigs positive for serum and the proportion positive by any of the swab sample types. The observed lack of PRRSV detection in swab samples when the *MCt*_*serum*_ is relatively high, is consistent with a similar study conducted on OS alone (23). This finding is also consistent with two previous studies that observed declining quantities of PRRSV in boar fluids and tissues following a decline in viremia (59, 73), further supporting the hypothesis that PRRSV in tissues is largely sourced from PRRSV-infected macrophages that get to the tissues from blood (74).

For this study, the volume of PBS used to elute fluids from the swab sticks was two milliliters; using that much diluent was aimed at ensuring a sufficient sample amount for testing, pooling, and storage (for future referencing). In practice, the RT-qPCR detection of PRRSV RNA in the swab samples could be further enhanced in real-world surveillance by taking up more piglet fluids on the swab stick and using a lesser volume of eluting solution ( ≤ 1 ml).

The relatively poor sensitivity of family oral fluids observed in this study is inconsistent with estimates from previous studies (75). This may be explained by the inadequate time allowed for sows and litters to interact with the sampling ropes. Considering the limited farm time available to sample piglets and organize the samples, sampling was conducted to optimize obtaining the needed number of piglet-level samples (the primary focus of this study).

Even though higher positivity rates in serum samples highlight the superiority of serum samples above other antemortem sample types in the RT-qPCR detection of PRRSV in *individual* piglets, population-based samples, however, have been proven to be more cost-efficient and practical in correctly assigning a PRRS status to a herd (10, 75, 76). Since the herd PRRSV status is most paramount to swine practitioners, it is no surprise that population-based samples are the most frequently submitted samples to U.S. veterinary diagnostic laboratories for PRRSV RT-qPCR investigations (8).

## 5. Conclusion

This study has successfully described and characterized PRRSV detection in ES, NS, and OS compared to serum. The effect of litter-level pooling of swab samples on PRRSV RNA detection was also successfully investigated.

The degree of viremia is a strong predictor of PRRSV detection in swab samples. There is a linear relationship between within-litter prevalence by serum and within-litter prevalence by other sample types. There was a near-perfect agreement between all piglet-level sample types but substantial to a near-perfect agreement for the litter-level sample types. When litter-pooled swabs are negative, the litter could be truly negative, or the proportion of viremic piglets in that litter is <30%. Litter-pooling of swab samples decreased sensitivity but increased specificity to 100%. Given the conditions of this study, the sensitivities and specificities of ES, NS, and OS are (0.83, ~1.00), (0.83, 0.99), and (0.75, 0.99), respectively. There was a ≥95% probability of PRRSV detection in ES-, OS-, and NS pools when the proportion of positive swab samples within the pools was ≥23%, ≥27%, and ≥26%, respectively.

ES, NS, and OS samples can be used for PRRSV surveillance in weaning age pigs. Practitioners can use the estimates of the diagnostic accuracies from this study to determine appropriate sample sizes.

## Data availability statement

The original contributions presented in the study are included in the article/Supplementary material, further inquiries can be directed to the corresponding author.

## Ethics statement

The animal study was reviewed and approved by Institutional Animal Care and Use Committee IACUC of Iowa State University, IA USA under protocol number IACUC-22-101.

## Author contributions

OO wrote the first draft of the manuscript. OO and DL conceptualized the study. OO, DL, GT, and GS reviewed the design of the study. OO, GC, RP, SJ, LP, IM, DM, MM-H, EM, and AP implemented the study, discussed the results, and reviewed the manuscript. PG and DL supervised the laboratory segment of the study and discussed the findings. All authors reviewed the study's preliminary findings and contributed to and reviewed this manuscript.
