# Supplementary material for: A cross-sectional assessment of PRRSV nucleic acid detection by RT-qPCR in serum, ear-vein blood swabs, nasal swabs, and oral swabs from weaning-age pigs under field conditions
Source: Front Vet Sci. 2023 Aug 10;10:1200376. doi: 10.3389/fvets.2023.1200376 (PMC10449646; doi:10.3389/fvets.2023.1200376)
Supplement: Supplementary file 1 [file Data_Sheet_1.pdf]

Supplemental Tables, Figure, and Text

Table S1. A matrix of "two-by-two" tables comparing the number of piglets testing PRRSV RT-qPCR positive or negative on serum samples, ear-vein swabs (ES), nasal swabs (NS), and oral swabs (OS) for each farm. "Neg" means "RT-qPCR negative", and "Pos" means "RT-qPCR positive.

Table S2. A matrix of "two-by-two" tables comparing the number of piglets testing PRRSV RT-qPCR positive or negative on serum samples, ear-vein swabs, nasal swabs, and oral swabs across all farms. "Neg" means "RT-qPCR negative", and "Pos" means "RT-qPCR positive.

Table S3. A matrix of cells showing crude agreement (first value within each cell), and Cohen's kappa value (second value within each cell) between serum samples, ear-vein swabs (ES), nasal swabs (NS), and oral swabs (OS).

Table S4. A matrix of two-by-two tables comparing RT-qPCR results of all litter-level sample types. "Neg" means "RT-qPCR negative", and "Pos" means "RT-qPCR positive."

Table S5. A matrix of cells showing crude agreement (first value within each cell), and Cohen's kappa value (second value within each cell) between pairs of the litter-level sample-types (ear-vein blood swab litter pools (ESp), nasal swab litter pools (NSp), oral swab litter pools (OSp), and family oral fluids (FOF)).

Table S6. A matrix of two-by-two tables comparing RT-qPCR results of all litter-level sample types (ear-vein blood swab litter pools (ESp), nasal swab litter pools (NSp), oral swab litter pools (OSp), and family oral fluids (FOF)) with the true PRRSV status of the litters (litters having ≥ viremic piglets). "Neg" means "RT-qPCR negative", and "Pos" means "RT-qPCR positive."

Table S7. The interpretation of the value ranges of the Cohen's Kappa statistic as reported by Landis and Koch (1977).

Table S8. The number of test samples by farm.

Table S9. An example of a two-by-two contingency table comparing the binary outcomes of two RT-qPCR tests.

Figure S1. The mean Ct per litter of swab samples (ear-vein blood swabs (top), nasal swabs (second from top), oral swabs (third from top), family oral fluids (bottom)) compared to the mean Ct per litter of serum.

Text S1. Formulas for calculating Cohen's kappa, sensitivity, specificity, positive predictive value, and negative predictive value.

Table S1. A matrix of "two-by-two" tables comparing the number of piglets testing PRRSV RT-qPCR positive or negative on serum samples, ear-vein swabs (ES), nasal swabs (NS), and oral swabs (OS) for each farm. "Neg" means "RT-qPCR negative", and "Pos" means "RT-qPCR positive."

		FARM 1			FARM 2			FARM 3		
SAMPLE TYPE		Serum			Serum			Serum		
	STATUS	Neg	Pos		Neg	Pos		Neg	Pos	
ES	Neg	219	2	**221**	172	3	**175**	176	11	**187**
	Pos	0	7	**7**	2	15	**17**	0	58	**58**
		**219**	**9**		**174**	**18**		**176**	**69**	
NS	Neg	220	2	**222**	170	5	**175**	176	17	**193**
	Pos	0	7	**7**	4	13	**17**	0	52	**52**
		**220**	**9**		**174**	**18**		**176**	**69**	
OS	Neg	220	2	**222**	169	3	**172**	174	11	**185**
	Pos	0	7	**7**	5	15	**20**	2	58	**60**
		**220**	**9**		**174**	**18**		**176**	**69**	

Table S2. A matrix of "two-by-two" tables comparing the number of piglets testing PRRSV RT-qPCR positive or negative on serum samples, ear-vein swabs, nasal swabs, and oral swabs across all farms. "Neg" means "RT-qPCR negative", and "Pos" means "RT-qPCR positive.

SAMPLE TYPE		Serum		ES		NS		OS	
	**STATUS**	**Neg**	**Pos**	**Neg**	**Pos**	**Neg**	**Pos**	**Neg**	**Pos**
**Serum**	**Neg**			567	2	566	4	563	7
	**Pos**			16	80	24	72	16	80
**ES**	**Neg**	567	16			580	3	573	10
	**Pos**	2	80			9	73	5	77
**NS**	**Neg**	566	24	580	9			575	15
	**Pos**	4	72	3	73			4	72
**OS**	**Neg**	563	16	573	5	575	4		
	**Pos**	7	80	10	77	15	72		

Table S3. Matrix of cells showing crude agreement (first value within each cell), and Cohen's kappa value (second value within each cell) between serum samples, ear-vein swabs (ES), nasal swabs (NS), and oral swabs (OS).

SAMPLE TYPE	Serum	ES	NS	OS
**Serum**		0.97, 0.88	0.96, 0.81	0.97, 0.85
**ES**	0.97, 0.88		0.98, 0.91	0.98, 0.90
**NS**	0.96, 0.81	0.98, 0.91		0.97, 0.87
**OS**	0.97, 0.85	0.98, 0.90	0.97, 0.87	

Table S4. A matrix of two-by-two tables comparing RT-qPCR results of all litter-level sample types. “Neg” means “RT-qPCR negative”, and “Pos” means “RT-qPCR positive.”

SAMPLE TYPE		ESp		NSp		OSp		FOF	
	**STATUS**	**Neg**	**Pos**	**Neg**	**Pos**	**Neg**	**Pos**	**Neg**	**Pos**
**ESp**	**Neg**			43	0	42	1	43	0
	**Pos**			1	11	1	11	5	7
**NSp**	**Neg**	43	1			42	2	44	0
	**Pos**	0	11			1	10	4	7
**OSp**	**Neg**	42	1	42	1			43	0
	**Pos**	1	11	2	10			5	7
**FOF**	**Neg**	43	5	44	4	43	5		
	**Pos**	0	7	0	7	0	7		

Table S5. A matrix of cells showing crude agreement (first value within each cell), and Cohen’s kappa value (second value within each cell) between pairs of the litter-level sample-types (ear-vein blood swab litter pools (ESp), nasal swab litter pools (NSp), oral swab litter pools (OSp), and family oral fluids (FOF)).

SAMPLE TYPE	ESp	NSp	OSp	FOF
**ESp**		0.98, 0.95	0.96, 0.89	0.91, 0.68
**NSp**	0.98, 0.95		0.95, 0.84	0.93, 0.74
**OSp**	0.96, 0.89	0.95, 0.84		0.91, 0.69
**FOF**	0.91, 0.68	0.93, 0.74	0.91, 0.69	

Table S6. A matrix of two-by-two tables comparing RT-qPCR results of all litter-level sample types (ear-vein blood swab litter pools (ESp), nasal swab litter pools (NSp), oral swab litter pools (OSp), and family oral fluids (FOF)) with the true PRRSV status of the litters (litters having ≥ viremic piglets). "Neg" means "RT-qPCR negative", and "Pos" means "RT-qPCR positive."

**SAMPLE TYPE**		**Litters with ≥ 1 viremic piglet**	
	**STATUS**	**Neg**	**Pos**
**ESp**	**Neg**	33	10
	**Pos**	0	12
**NSp**	**Neg**	33	11
	**Pos**	0	11
**OSp**	**Neg**	33	10
	**Pos**	0	12
**FOF**	**Neg**	33	15
	**Pos**	0	7

Table S7. The interpretation of the value ranges of the Cohen's Kappa statistic as reported by Landis and Koch (1977).

**Cohen's kappa value**	**Interpretation**
< 0.00	Poor agreement
Between 0.00 and 0.20	Slight agreement
Between 0.21 and 0.40 (inclusive)	Fair agreement
Between 0.41 and 0.60 (inclusive	Moderate agreement
Between 0.61 and 0.80 (inclusive)	Substantial agreement
Between 0.81 and 1.00 (inclusive)	Almost perfect agreement

Table S8. The number of test samples by farm

	Piglet-level samples					Litter-level samples					
**Farm**	ES	NS	OS	Srm	**Subtotal**	ES-pool	FOF	NS-pool	OS-pool	**Subtotal**	**Total**
**1**	228*	229	229	229	**915**	20	20	20	20	**80**	**995**
**2**	192	192	192	192	**768**	15	15	15	15	**60**	**828**
**3**	245	245	245	245	**979**	20	20	20	20	**80**	**1059**
**Total**	**665**	**666**	**666**	**666**	**2662**	**55**	**55**	**55**	**55**	**220**	**2882**

**The ES sample from one piglet was lost on-farm (fell through the slats).*

From left to right: ear-vein blood swabs (ES), nasal swabs (NS), oral swabs (OS), serum (Srm), ear-vein blood swab pools (ES-pool), family oral fluids (FOF), nasal swab pools (NS-pool), and oral swab pools (OS-pools).

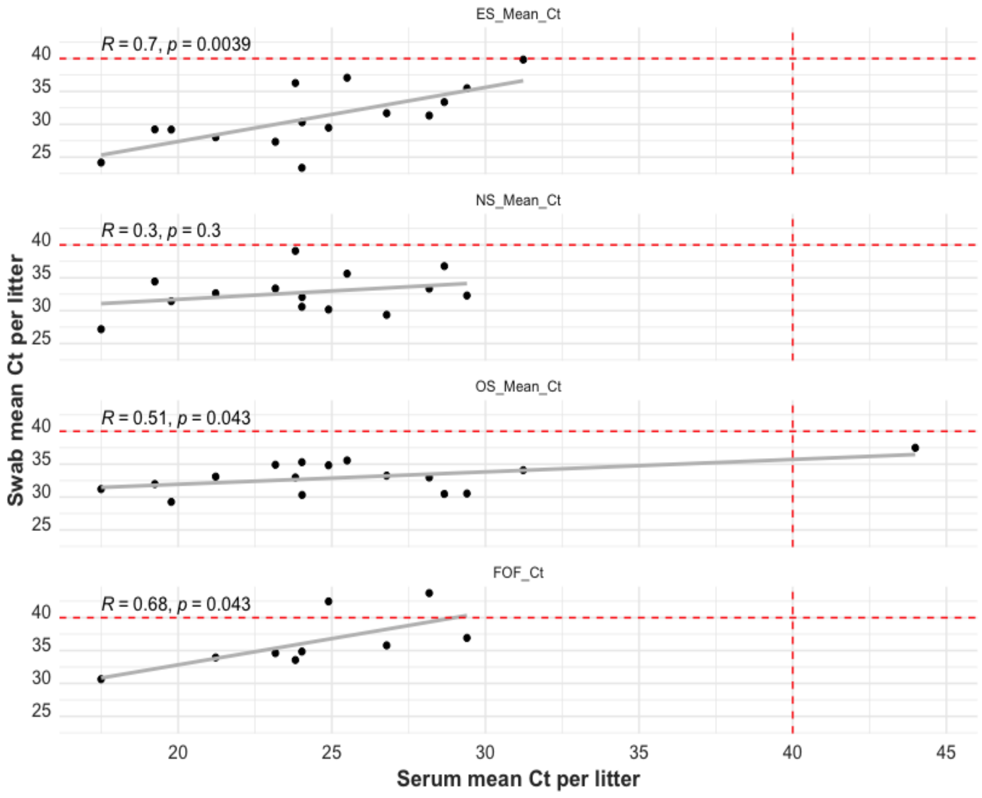

Figure S1. The mean Ct per litter of swab samples (ear-vein blood swabs (top), nasal swabs (second from top), oral swabs (third from top), family oral fluids (bottom)) compared to the mean Ct per litter of serum.

Table S9. An example of a two-by-two contingency table comparing the binary outcomes of two RT-qPCR tests.

		**Reference sample or test**		
		**Neg**	**Pos**	
**New sample or test**	**Neg**	α	β	N_-
	**Pos**	δ	ε	N_+
		R_-	R_+	

Text S1. Formulas for calculating crude agreement, Cohen's kappa, sensitivity, specificity, positive predictive value, and negative predictive value.

The crude agreement is calculated as: $\frac{\alpha + \varepsilon}{\alpha + \beta + \delta + \varepsilon}$ (1)

The Cohen's kappa statistic (C_k) is calculated as:

$$\frac{\frac{\alpha + \varepsilon}{\alpha + \beta + \delta + \varepsilon} - \left(\left(\left(\frac{N_+}{\alpha + \beta + \delta + \varepsilon}\right) * \left(\frac{R_+}{\alpha + \beta + \delta + \varepsilon}\right)\right) + \left(\left(\frac{N_-}{\alpha + \beta + \delta + \varepsilon}\right) * \left(\frac{R_-}{\alpha + \beta + \delta + \varepsilon}\right)\right)\right)}{1 - \left(\left(\left(\frac{N_+}{\alpha + \beta + \delta + \varepsilon}\right) * \left(\frac{R_+}{\alpha + \beta + \delta + \varepsilon}\right)\right) + \left(\left(\frac{N_-}{\alpha + \beta + \delta + \varepsilon}\right) * \left(\frac{R_-}{\alpha + \beta + \delta + \varepsilon}\right)\right)\right)} \quad (2)$$

Sensitivity $= \frac{\varepsilon}{R_+}$ (3)

Specificity $= \frac{\alpha}{R_-}$ (4)

Positive predictive value $= \frac{\varepsilon}{N_+}$ (5)

Negative predictive value $= \frac{\alpha}{N_-}$ (6)
